# Phase Measuring Deflectometry for Wafer Thin-Film Stress Mapping

**DOI:** 10.3390/s25247668

**Published:** 2025-12-18

**Authors:** Yang Gao, Xinjun Wan, Kunying Hsin, Jiaqing Tao, Zhuoyi Yin, Fujun Yang

**Affiliations:** 1 Jiangsu Key Laboratory of Engineering Mechanics, Southeast University, Nanjing 210096, China; gaoylamb@163.com; 2 Shanghai Key Laboratory of Modern Optical System, University of Shanghai for Science and Technology, Shanghai 200093, China; xinjun.wan@usst.edu.cn (X.W.); 13262631579@163.com (J.T.); 3 Suzhou Raphael Optech Co., Ltd., Suzhou 215400, China; xinky@raphaeloptech.com; 4 School of Science, Nanjing University of Science and Technology, Nanjing 211189, China; yinzhuoyi@njust.edu.cn; 5 Hubei Key Laboratory of Electronic Manufacturing and Packaging Integration, Wuhan University, Wuhan 430072, China

**Keywords:** PMD, wafer, thin-film stress

## Abstract

Wafer-level thin-film stress measurement is essential for reliable semiconductor fabrication. However, existing techniques present limitations in practice. Interferometry achieves high precision but at a cost that becomes prohibitive for large wafers. Meanwhile laser-scanning systems are more affordable but can only provide sparse data points. This work develops a phase-measuring deflectometry (PMD) system to bridge this gap and deliver a full-field solution for wafer stress mapping. The implementation addresses three key challenges in adapting PMD. First, screen positioning and orientation are refined using an inverse bundle-adjustment approach, which performs multi-parameter optimization without re-optimizing the camera model and simultaneously uses residuals to quantify screen deformation. Second, a backward-propagation ray-tracing framework benchmarks two iterative strategies to resolve the slope-height ambiguity which is a fundamental challenge in PMD caused by the absence of a fixed optical center on the source side. The reprojection constraint strategy is selected for its superior convergence precision. Third, this strategy is integrated with regional wavefront reconstruction based on Hermite interpolation to effectively eliminate edge artifacts. Experimental results demonstrate a peak-to-valley error in the reconstructed topography of 0.48 µm for a spherical mirror with a radius of 500 mm. The practical utility of the system is confirmed through curvature mapping of a 12-inch patterned wafer and further validated by stress measurements on an 8-inch bare wafer, which show less than 5% deviation from industry-standard instrumentation. These results validate the proposed PMD method as an accurate and cost-effective approach for production-scale thin-film stress inspection.

## 1. Introduction

Three-dimensional reconstruction of specular surfaces has become indispensable in the microelectronics and aerospace industries, with applications spanning free-form mirrors [1], astronomical telescopes [2], and wafers inspection [3,4]. In microelectronics, packaging technology has progressively shifted to the wafer level, while 12-inch SiC substrates have entered high-volume production for power devices [5,6]. These developments are largely driven by the need to reduce cost per chip and enhance computational performance through larger wafer sizes, particularly as Moore’s Law approaches its physical limits. However, this evolution toward larger wafers and advanced packaging architectures introduces dual challenges, with increasingly complex thermal deformation and aggravated warpage being the most prominent [7]. These issues primarily stem from the coefficients of thermal expansion (CTE) mismatch, film non-uniformity during multilayer deposition, and intrinsic substrate stress (e.g., from lattice defects) [8]. These components collectively manifest as thin-film stress, which severely degrades the yield of critical processes such as photolithography [9]. Therefore, developing techniques capable of accurately characterizing full-field thin-film stress is essential for successful implementation of large-wafer-scale advanced packaging [10,11]. Central to this effort is the Stoney equation, which enables extraction of thin-film stress from surface curvature when material properties are known [12]. Thus, obtaining a two-dimensional curvature distribution of the wafer surface using techniques such as ULTRATECH CGS becomes critically important for fundamentally mitigating the risk of chip failure [13]. However, this inspection requirement faces two challenges. First, traditional semiconductor inspection methods such as the shadow moiré technique are unsuitable for the mirror-like surface of wafers [14]. While established techniques like stereo digital image correlation (DIC) and phase-measuring profilometry (PMP) are capable of real-time 3D reconstruction, they still require surface spray coating, a practice that is prohibited in semiconductor manufacturing [15,16]. Second, while interferometry and patterned wafer geometry inspection systems (e.g., KLA PWG5) provide high precision, these systems require large-aperture aspheric lenses and mirrors, which entails a prohibitive cost that limits their practical deployment for large-sized wafers [17,18,19]. Additionally, current laser-scanning or area-array reflection techniques (e.g., KSA MOS) yield only sparse data, relying on curve fitting to approximate the full-field distribution [20,21]. Recently, phase-measuring deflectometry (PMD) has emerged as a promising alternative, offering high speed, high accuracy, and a wide measurement range for specular-surface characterization and stress analysis [22,23,24,25]. In PMD, structured-light fringe patterns are displayed on a screen and reflected by the specular surface under test (SUT). The reflected patterns, which are distorted according to the surface geometry, are captured by a camera. Following system calibration, the surface slopes can be computed, enabling the three-dimensional topography to be reconstructed via numerical integration.

The determination of thin-film stress within the PMD framework entails a system calibration comprising two fundamental components: intrinsic camera calibration and extrinsic geometric calibration [26]. The intrinsic calibration estimates the camera’s internal parameters, including focal length, principal-point offset, and distortion coefficients. This is a well-established procedure in computer vision, with Zhang’s method being the most widely adopted approach [27]. The extrinsic calibration establishes the spatial relationships between the screen, reference mirror, and camera [28], presenting a particular challenge since the screen lies outside the camera’s field of view, making it inherently invisible and requiring specialized calibration procedures [29]. Existing extrinsic calibration methods can be categorized into three distinct approaches based on their complexity and hardware requirements. The first method utilizes a reference plane mirror with precision markers placed at the specimen position [30], where coordinates are pre-determined using stereoscopic camera systems to establish a precise correspondence between screen pixels, mirror reflection points, and camera pixels. The second approach employs laser trackers or optical coordinate measuring machines (CMMs) to directly capture the spatial coordinates of both the camera aperture center and screen pixels, achieving absolute positioning through sequential measurements [31]. While both methods provide high accuracy, they require expensive, bulky metrology equipment and involve time-consuming operations that hinder practical deployment in production environments. Consequently, a third calibration strategy has been developed where a standard plane mirror without reference points is sequentially positioned in at least three distinct orientations [32]. This approach significantly reduces system complexity and cost while maintaining flexibility in mirror placement, making it particularly suitable for wafer-level thin-film stress measurement applications.

However, in the PMD calibration process, the true limit is not determined by the initial values, but rather by the subsequent global optimization. The bundle adjustment (BA) method is naturally used to simultaneously refine the camera’s intrinsic parameters, the screen’s extrinsic parameters, and the mirror poses through 2D reprojection (Appendix A) [33]. The multi-pose BA approach requires only reflection transformation constraints. In case of a single-pose or spherical mirror, however, the mirror geometry (position, dimensions, curvature) must be incorporated as a spatial invariant in BA, leading to a sharp increase in model complexity [34]. In addition, the results obtained in this manner only indicate that the fitting of parameters has high precision, but this does not guarantee that the model is more consistent with the actual physical system [35,36]. For instance, the calculated lens distortion no longer exhibits a radial distribution. Recently, visual ray adjustments break free from the classical pinhole and distortion-based camera framework by fitting discrete vision rays [37]. However, both approaches inherently deviate from the classical camera model. In contrast, this work retains the classical camera model and attributes reflection errors primarily to uncertainties in the mirror position. These errors will propagate into the camera model, causing the intrinsic parameters to deviate from their true values and leading the parameter optimization process to become trapped in local minima. Therefore, this paper adopts a reverse optical path optimization approach, that is, fixing the calibrated camera model while treating only the screen’s extrinsic parameters as variables and performing direct back-propagation. Simultaneously, the optimization residuals are leveraged to compensate for screen deformation, enhancing the physical faithfulness of the calibration.

Furthermore, since PMD employs a display screen as its light source, it lacks a defined optical center. Therefore, conventional stereo triangulation is not applicable, introducing the inherent slope-height ambiguity. Stereo PMD [38], direct PMD [39], and polarization PMD techniques [40] have made considerable contributions to this issue. Among these, the seed-point-based iterative strategy has emerged as the mainstream solution due to its favorable balance between accuracy and stability [41]. Iterative strategies are categorized into two mutually inverse types, including optical-center constraints (OC) and reprojection constraints (RC). The former proceeds along pinhole rays, but perspective transformation causes the coordinate system to form irregular quadrilaterals, disrupting the ideal grid for regional wavefront integration and amplifying errors [42]. The latter pre-sets a uniform grid, thus avoiding geometric distortion, but lacks theoretical precision analysis. Therefore, this paper conducts simulations within a PMD ray-tracing framework to quantitatively compare the ultimate limits of accuracy of both strategies.

Furthermore, two-dimensional integration fundamentally involves solving the Poisson equation. Commonly used integration approaches in PMD include modal wavefront integration, piecewise reconstruction, and zonal wavefront reconstruction [43,44]. For wafer stress measurement, it is crucial to preserve mid-to-high-frequency surface details, as they directly reflect defects introduced by manufacturing processes. However, modal and piecewise methods tend to smooth out these critical components. In contrast, the Southwell zonal wavefront integration method maintains the computational independence of individual height points due to its inherent grid-based structure. However, the Southwell method is highly sensitive to boundary conditions. Discontinuities at the boundaries amplify high-frequency errors, leading to edge spikes and noise [45]. To eliminate these artifacts, this paper introduces Hermite-spline-based zonal wavefront integration. By preserving first-order differentiability and second-order continuity along edges via Hermite interpolation, the method effectively mitigates boundary-induced errors, significantly improves the accuracy of 3D topography reconstruction, and enhances robustness in the presence of complex scenes and locally missing data.

This paper is organized as follows. Section 2 introduces the underlying principle of thin-film stress measurement using PMD. Section 3 details the PMD calibration methodology. Section 4 provides a comparative analysis of two iterative reconstruction strategies. Section 5 addresses the elimination of edge artifacts. Section 6 presents experimental results demonstrating the performance of the PMD-based wafer thin-film stress detection system. Section 7 concludes the paper with a summary of findings.

## 2. Principle

**Figure 1:**
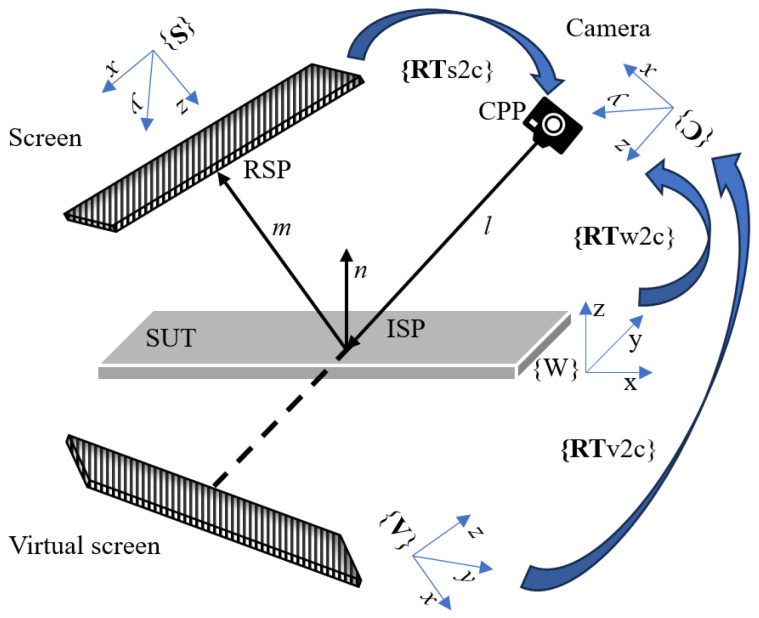
PMD model and the ambiguity of the slope and height.

PMD is a backward trace analysis and Figure 1 illustrates the PMD model. The screen and camera coordinate frames are defined as **{S}** and **{C}**, respectively. **{V}** denotes the virtual screen, the virtual image of the real screen. **R** and **T** represent the rotation matrix and translation vector, respectively. Subscripts indicate the transformation from the source coordinate to the target coordinate. For example, **Rw2c** is the rotation matrix from the world frame **{W}** to the camera frame **{C}**, and similarly for other transformations.

Based on differential geometry, the slope can be established, as shown in Equation (1) [46].

(1) $$\frac{\partial z}{\partial x}=\frac{\frac{x_{t}-x_{c}}{d_{t}^{c}}-\frac{x_{t}-x_{s}}{d_{t}^{s}}}{\frac{z_{c}-z\left(x_{t},y_{t}\right)}{d_{t}^{c}}+\frac{z_{s}-z\left(x_{t},y_{t}\right)}{d_{t}^{s}}},\frac{\partial z}{\partial y}=\frac{\frac{y_{t}-y_{c}}{d_{t}^{c}}-\frac{y_{t}-y_{s}}{d_{t}^{s}}}{\frac{z_{c}-z\left(x_{t},y_{t}\right)}{d_{t}^{c}}+\frac{z_{s}-z\left(x_{t},y_{t}\right)}{d_{t}^{s}}}$$

where $\left[x_{t},y_{t},z_{t}\right]$ represents the SUT, $\left[x_{c},y_{c},z_{c}\right]$ represents the optical center O, and $\left[x_{s},y_{s},z_{s}\right]$ represents the screen points. $\left[d_{t}^{c},d_{t}^{s}\right]$ represents the distance from the SUT to O and the screen pints.

**Figure 2:**
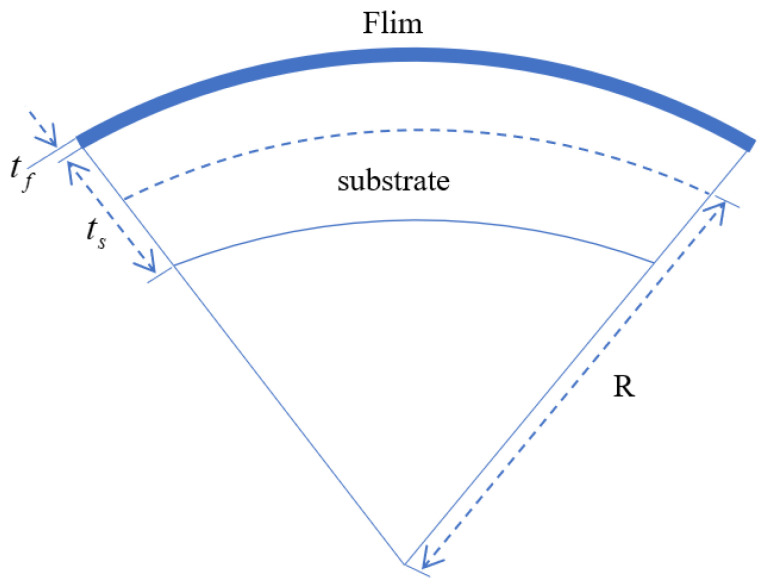
The Stoney model for thin-film stress.

The Stoney formula relates the curvature change of a substrate deposited thin film, as shown in Figure 2. It is widely used in coating technology and semiconductor processing. Provided the thicknesses and elastic properties of the film and substrate, the stress is obtained in Equation (2) once the radius *R* is determined.

(2) $$\sigma =\frac{E_{s}{t_{s}}^{2}}{6\left(1-v_{s}\right)t_{f}R}$$

where σ represents residual stress in the film, $E_{s}$, $v_{s}$ represent Young’s modulus and Poisson’s ratio of the substrate, $t_{s}$ represents substrate thickness, $t_{f}$ represents film thickness, *R* represents radius of curvature of the substrate. With the surface topography measured by PMD, *R* can be calculated from differential geometry.

## 3. Global Optimization of Calibration

**Figure 3:**
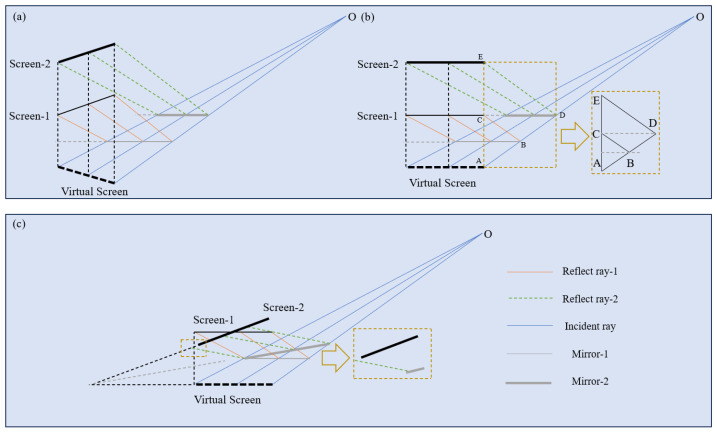
Solution domain of the optical path induced by screen extrinsics and mirror poses. (**a**,**b**) ambiguous ray paths arising from the combination of screen translation vectors and mirror poses; (**c**) unique ray path determined by the screen rotation matrix and mirror pose.

As shown in Figure 3, variations in either the translation vector or the rotation matrix of the screen modify the optical path. In the case of translation, Figure 3a depicts the propagation path of a pinhole ray under two screen positions differing only in translation. Since the mirror pose (translational and rotational position) is unknown, an arbitrary plane can be intercepted along the optical path to represent the mirror. For clarity and without loss of generality, a vertically oriented plane normal is assumed, as shown in Figure 3b. The similarity of triangles ABC and ADE indicates that multiple mirror poses can satisfy the ray-tracing condition from the camera to the screen. In contrast, for the rotation matrix, the mapping between the screen and the mirror pose is unique, as demonstrated in Figure 3c. However, the translation vector introduces ambiguity in the optical path, which can lead to convergence to local minima during optimization. Therefore, multi-pose optimization is essential to resolve these ambiguities, as it does not require prior knowledge of the mirror’s geometry or position.

**Figure 4:**
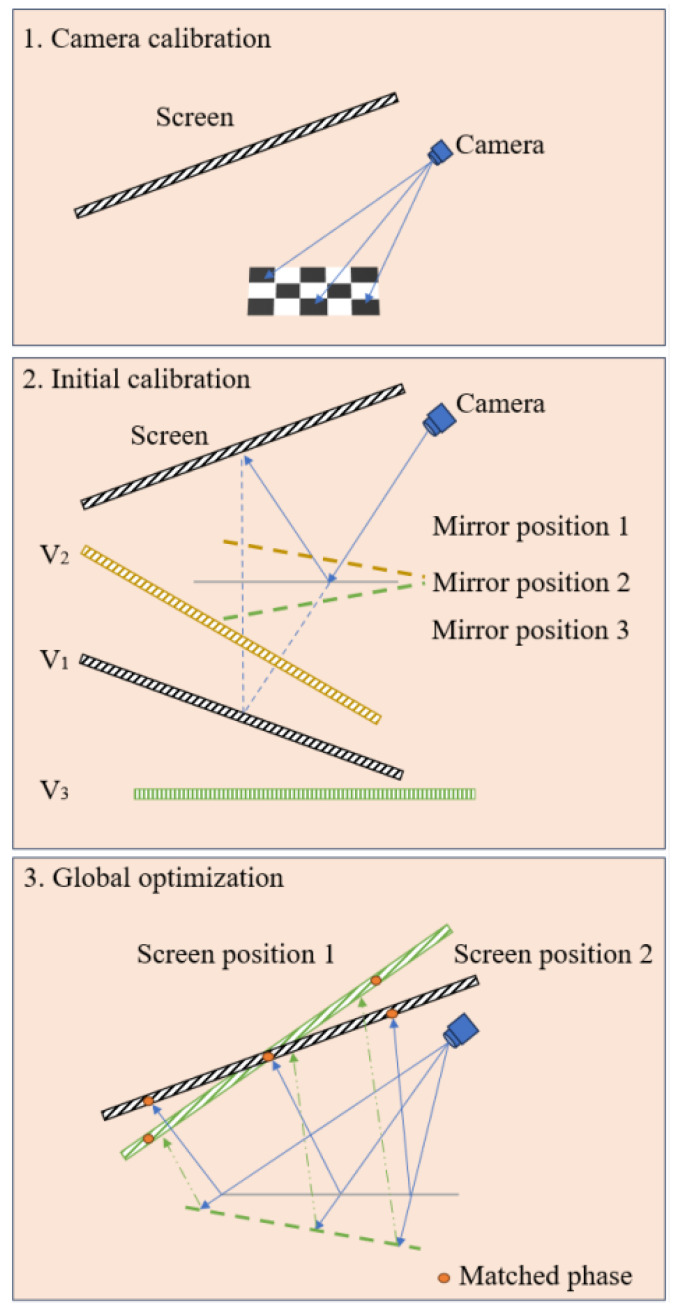
Global optimization workflow for backward-path calibration.

However, conventional multi-pose reprojection methods assign all fitting residuals to camera distortion, resulting in a distortion distribution that significantly deviates from the typical radial model. To avoid solutions that are mathematically feasible yet physically implausible, we employ a backward ray-tracing model. This method preserves the calibrated camera intrinsics and optimizes solely the screen pose. The overall procedure is illustrated in Figure 4.

Step 1: calibrate the camera with a checkerboard.

Step 2: translate the planar mirror three times to obtain the initial extrinsic parameters $R_{S2C}$ and $T_{S2C}$.

Step 3: refine the parameters by inverse rays tracing.

In step 3, every valid pixel is assigned a pinhole ray, as expressed in Equation (3). This ray is defined by its direction vector ${\left[d_{x},d_{y},d_{z}\right]}^{T}$ and the coordinate origin (the optical center).

(3) $$\left[\begin{array}{c} d_{x}\\ d_{y}\\ d_{z} \end{array}\right]=K^{-1}\left[\begin{array}{c} u\\ v\\ 1 \end{array}\right]$$

According to the Householder transformation, the virtual screen is computed in Equation (4) as the reflection of the real screen about that mirror plane [47].

(4) $$RT_{S}=(I-2n_{C}•{n_{C}}^{T})RT_{V}$$

where $n_{C}$ represents the normal of the mirror, which can be calculated from step 2. Thus, the normal vector of the virtual screen plane is determined by the translation vector $T_{V2C}$ and the rotation matrix $R_{V2C}$.

(5) $$L=\frac{1}{N}\sum_{i=1}^{N}\left[{\left(x_{i}-x_{i}^{\left(p\right)}\right)}^{2}+{\left(y_{i}-y_{i}^{\left(p\right)}\right)}^{2}\right]$$

The calibration parameters are globally optimized by minimizing the mean squared error function defined in Equation (5), which quantifies the total squared difference across all *N* data points between the intersection point $\left(x_{i},y_{i}\right)$ of each pinhole ray with the virtual screen plane and its corresponding phase-matched point $\left(x_{i}^{p},y_{i}^{p}\right)$ on that plane.

**Figure 5:**
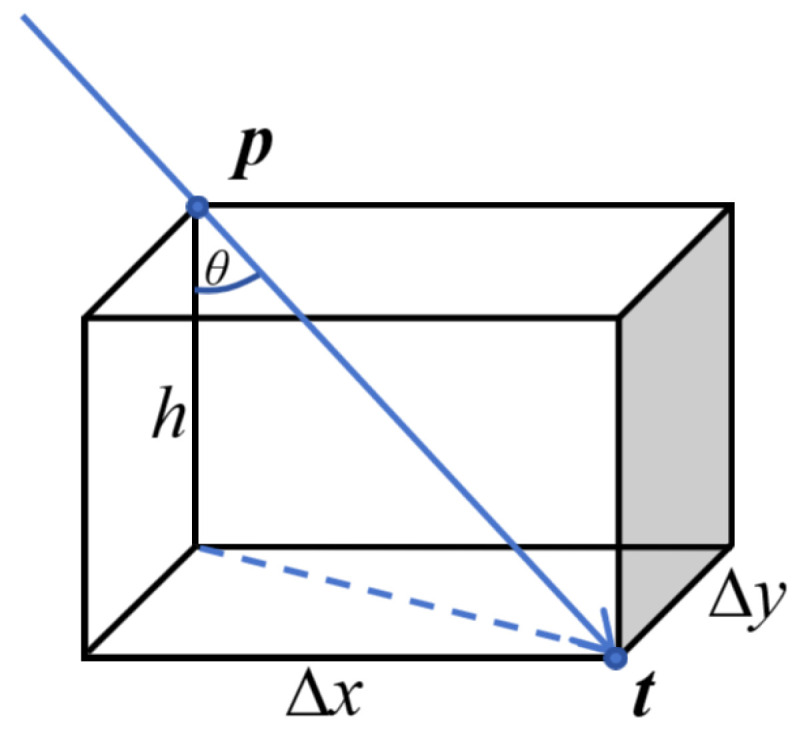
Pre-defined relationship between optimization residuals and screen out-of-plane deformation.

Furthermore, we assume a relationship between screen bending and the residual, as shown in Figure 5. The pinhole ray is assumed to strike the actual curved screen at point ***p***, while its intersection with the fitted plane is denoted as ***t***.

This configuration forms a spatial triangle. The sagitta of every sampled screen point is then given by Equation (6).

(6) $$z=\frac{\sqrt{\Delta x^{2}+\Delta y^{2}}}{\tan\theta }$$

where θ is the angle between the incident ray and the screen normal. Collecting all residuals and fitting them yields the deformed screen surface. This is a simplified model, since glass refraction is ignored and its validity will be examined in the experiments section.

## 4. Iteration Strategies

Among the various approaches for mitigating the inherent slope-height ambiguity in PMD, the seed-point-based iterative scheme is the most widely adopted. Its two mainstream variants are the optical-center constraint (OC) and the reprojection constraint (RC). Although both are routinely employed, the theoretical accuracies of the two variants have rarely been compared.

### 4.1. OC and RC Strategies

For convenience, this paper defines these four points. The camera pixel point (CPP) on the focal plane emits a pinhole ray l. The intersection of the pinhole ray with the actual SUT surface is called the incident slope point (ISP), and the intersection with the computed surface is called the incident height point (IHP). The intersection of the reflected ray m with the screen is called the reflected screen point (RSP).

**Figure 6:**
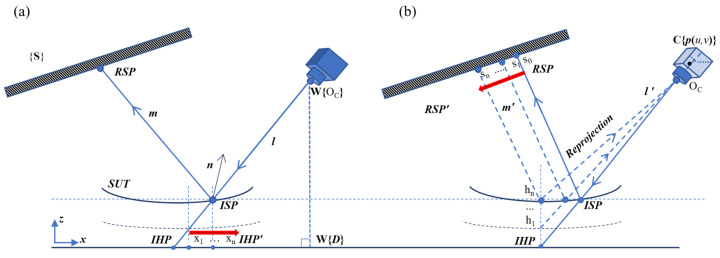
Iterative strategies: (**a**) the optical-center constraint (OC) strategy; (**b**) the reprojection constraint (RC) strategy.

The steps of the OC iterative reconstruction are as follows. First, the initial height is obtained and substituted into Equation (1) to calculate the slope data. Then, integration is performed to updated the height, and the OC constraint is used to form the triangle. By directly utilizing the OC constraint in Equation (7), we can continuously update the x-y coordinates. The process of the updated IHP is shown by the red arrows in Figure 6a.

(7) $$\begin{array}{c} x_{n}=\left(z_{n}-z_{c}\right)\frac{x_{l}}{z_{l}}+x_{c},\\ y_{n}=\left(z_{n}-z_{c}\right)\frac{y_{l}}{z_{l}}+y_{c} \end{array}$$

**Figure 7:**
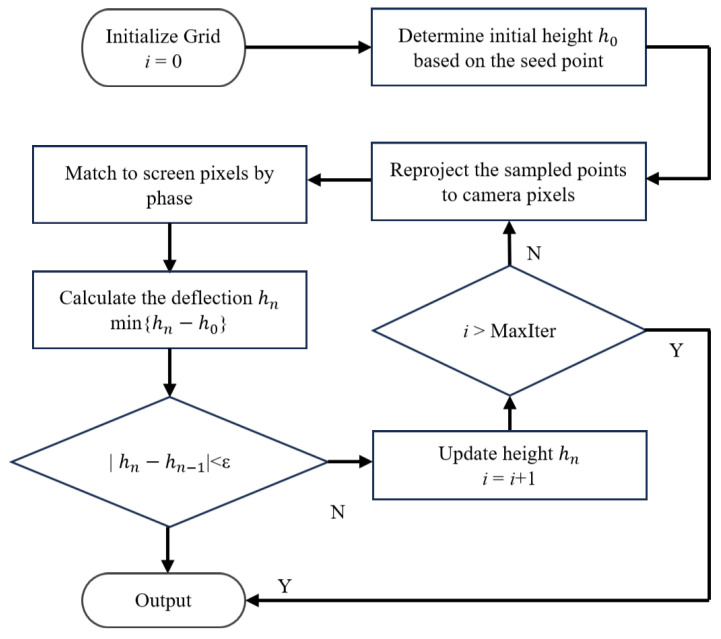
The flowchart of the RC iterative strategy.

RC iterative process begins by redefining a uniform grid for the SUT. The grid range is determined according to the boundary of the SUT. We then use a specified grid spacing to resample the SUT, and the height of a seed point is set as the initial plane. Since the grid is customized to remain fixed, this iterative process focuses on finding the corresponding screen points ISP, and this process performs with reprojection and phase matching, as shown by the red arrows in Figure 6b. It can be seen from Figure 6b that once the seed point is designated as the initial IHP plane, the slope is calculated. The height is then reconstructed through integration, so that the IHP is immediately updated. Hence, the 3D coordinates of the updated IHP are reprojected into the camera to obtain the new CPP. By using the phase matching in Equation (8), the RSP moves to the new position.

(8) $$\begin{array}{c} \varphi \left(x_{n}\right)=\varphi \left\{K_{1}\left(R{\left[x,y,z_{n}\right]}^{T}+T\right)\right\},\\ \varphi \left(y_{n}\right)=\varphi \left\{K_{2}\left(R{\left[x,y,z_{n}\right]}^{T}+T\right)\right\} \end{array}$$

where $K_{1}$ represents the first row of the intrinsic matrix. As the iteration progresses, the CPP, IHP, and RSP, will gradually pair. The flowchart is shown in Figure 7.

### 4.2. Simulation

To compare the accuracy of the two strategies, we developed a simulation model. The camera center position represented using Euler angles, and translation vector is ${\left[180,20,400\right]}^{T}$ and rotation matrix is ${\left[180,24,4\right]}^{T}$ mm. The focal length is defined as 12 mm, the pixel pitch is 3.5 µm, and the resolution is 720 × 720 pixels. The screen is set as a plane z=300 mm, and the initial height is set to z=0. The intersection points are determined using the Newton method, as shown in Equation (9).

(9) $$\begin{array}{c} {\left[x^{\prime },y^{\prime },z^{\prime }\right]}^{T}={\left[x,y,z\right]}^{T}+t{\left[l,m,n\right]}^{T},\\ t=\Delta z/n \end{array}$$

where ${\left[l,m,n\right]}^{T}$ represents the direction, Δz is the difference of height, and the accuracy is set to $10^{-9}\;\mathrm{mm}$.

**Figure 8:**
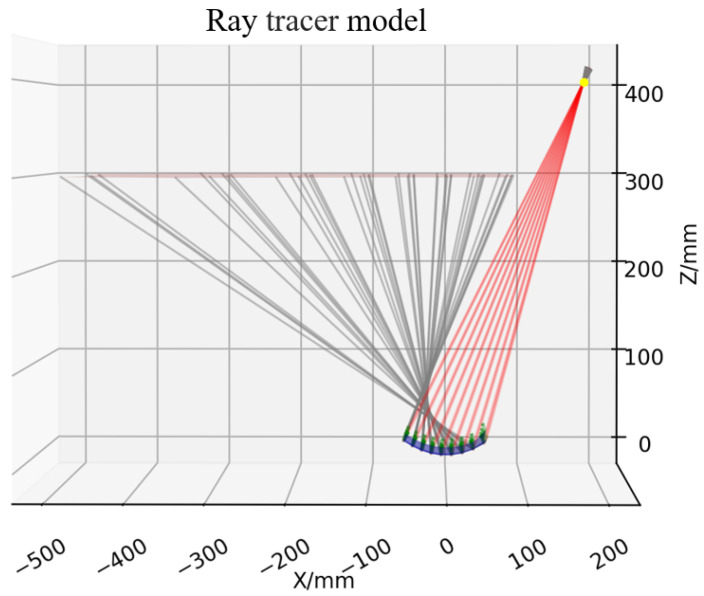
Simulation schematic.

Figure 8 shows the tracing model, with 15 × 15 rays in the effective region. Two types of curved surfaces are defined on the SUT. The spherical surface size is 70 × 50 mm, with a diameter of 150 mm and a curvature radius of 250 mm. The free-form surface is given by Equation (10), with dimensions the same as that of the spherical surface.

(10) z=3cos(x)+5sin(y)

**Figure 9:**
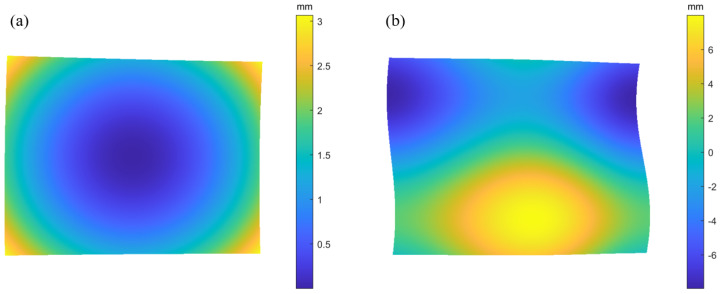
Simulated surfaces of the OC strategy: (**a**) spherical surface; (**b**) freeform surface.

**Figure 10:**
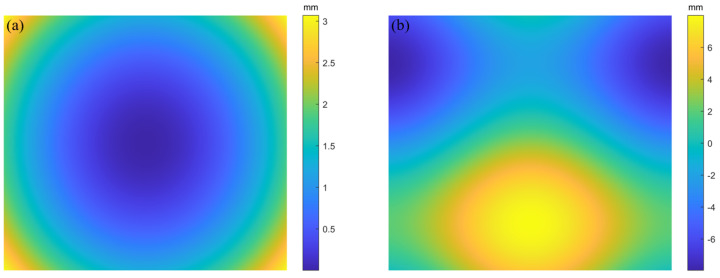
Simulated surfaces of the RC strategy: (**a**) spherical surface; (**b**) freeform surface.

Figure 9a,b and Figure 10a,b show the reference sampling points for the OC method and RC method, respectively.

These sampling points have true values and can be used as a benchmark for verification with subsequent reconstruction results. The number of valid reference sampling points for both methods is 200 × 300.

The reconstruction is then carried out using the Southwell quadrilateral grid integration method [48]. It should be noted that edge artefacts are disregarded here. These issues will be discussed in the following subsection.

**Figure 11:**
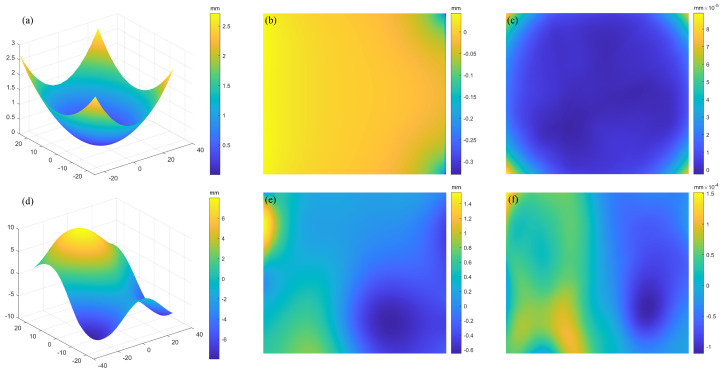
Reconstruction error of the RC method: (**a**,**d**) reconstruction results; (**b**,**e**) residuals of the spherical and freeform surface at the first round iteration; (**c**,**f**) residuals of the spherical and freeform surface at the fourth round iteration.

The RC simulation results of the spherical SUT are shown in Figure 11. It can be seen from Figure 11a that the initial reconstructed profile peak-to-valley (PV) error 373.1 µm reflects the impact of the ambiguity. The PV value for each iteration is displayed in the Table 1. It can be observed that, after 4 iterations, the PV error shrinks to 0.0916 µm because the error for PMD is much less sensitive to height than it is to slope. Once the system obtains the relative prediction surface, its accuracy will be significantly improved. The reconstructed free-form SUT are shown in Figure 11d. The initial reconstruction PV = 2206.7 µm. After 4 rounds of iterations, the error drops to PV = 0.2627 µm. It can be seen that the integral reconstruction accuracy is affected by the complexity of the SUT.

**Figure 12:**
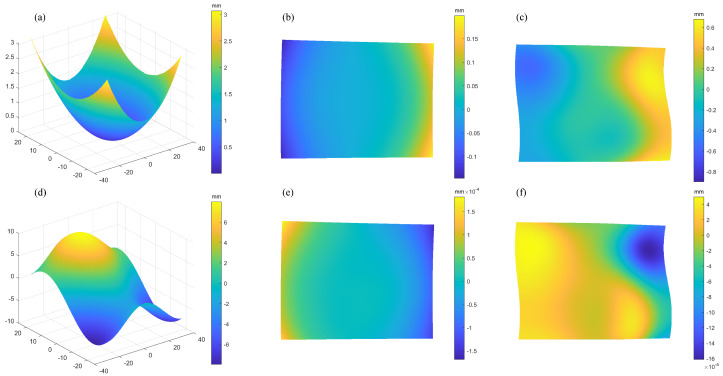
Reconstruction error of the OC method: (**a**,**d**) reconstruction results, (**b**,**e**) residuals of the spherical and freeform surface at the first round iteration. (**c**,**f**) residuals of the spherical and freeform surface at the fourth round iteration.

The OC simulation results of the spherical SUT are shown in Figure 12. It can be seen from Table 1 that it achieves better initial calculation accuracy. However, as the iterations proceed, the reconstruction accuracy is limited in improvement. After four rounds of iterations, the PV error of the spherical mirror is 0.348 µm in Figure 12c, while the error of the freeform surface is 2.09 µm in Figure 12f. This represents an accuracy loss of approximately one order of magnitude compared to the RC strategy.

It is evident that both the RC and OC methods converge in a comparable number of iterations. However, the RC strategy achieves higher reconstruction accuracy. Meanwhile, the RC strategy utilizes a custom-defined coordinate grid that preserves data range and spatial coordinates throughout deformation processing. These capabilities ensure superior data authenticity and accuracy in critical deformation calculations for wafer thin-film stress measurement, making it the preferred choice for wafer inspection.

## 5. Edge Artifact Suppression

**Figure 13:**
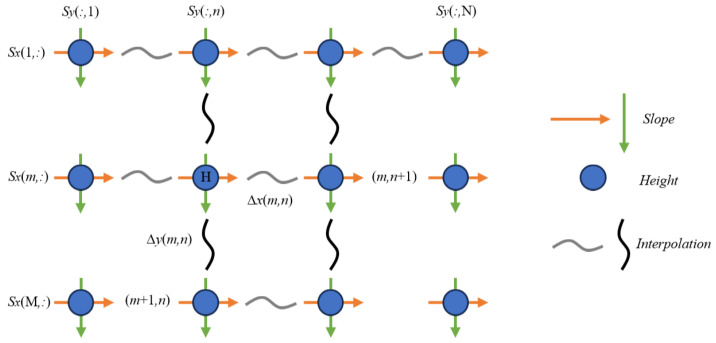
The geometry of Southwell.

Iterative reconstruction suffers from a critical flaw because spikes appear at the edges when zonal wavefront integration becomes discontinuous across the boundaries. Spline interpolation can alleviate this problem [49]. To further enhance the integration accuracy and applicability, we use Hermite zonal wavefront integration, which maintains the highest reconstruction precision even when the data contains numerous boundaries discontinuities. The slope field is interpolated using Hermite splines so as to express height differences between points, as shown in Figure 13.

Height differences between neighboring points are computed through total differentials. The coefficients of the segmented polynomial at each grid point are obtained by solving the Hermite interpolation system under given constraints as Equation (11).

(11) $$\begin{array}{c} z_{m,n+1}-z_{m,n}=\sum_{k=0}^{3}\frac{1}{k+1}c_{m,n,k}^{x}\Delta x_{m,n}^{k+1},\\ z_{m+1,n}-z_{m,n}=\sum_{k=0}^{3}\frac{1}{k+1}c_{m,n,k}^{y}\Delta y_{m,n}^{y+1} \end{array}$$

where $\Delta x_{m,n}=x_{m,n+1}-x_{m,n}$, $\Delta y_{m,n}=y_{m,n+1}-y_{m,n}$ is the x and y step at matrix position (m,n) as shown in Figure 13. $c_{m,n,k}^{x}$, $c_{m,n,k}^{y}$ is the coefficient of the kth order segmented polynomial starting at (m,n), which can be determined by solving the Hermite interpolation to satisfy the constraints, as shown in Equation (12).

(12) $$\left\{\begin{array}{c} H\left(x_{m,n}\right)=s_{m,n}^{x}\\ H\left(x_{m,n+1}\right)=s_{m,n+1}^{x}\\ H^{\prime }\left(x_{m,n}\right)={\left(s_{m,n}^{x}\right)}^{\prime }\\ H^{\prime }\left(x_{m,n+1}\right)={\left(s_{m,n+1}^{x}\right)}^{\prime } \end{array}\right.$$

The SUT is defined analytically by Equation (13) [48].

(13) $$z=\cos\left(2\pi x^{2}/3000\right)\times \sin\left(2\pi y^{2}/3002\right)$$

Four integration methods are compared: Traditional Finite-difference-based Least-squares Integration (TFLI), Higher-order Finite-difference-based Least-squares Integration (HFLI), Spline-based Least-squares Integration (SLI), and the Spline-based Least-squares Integration using Hermite interpolation (SLIH).

**Figure 14:**
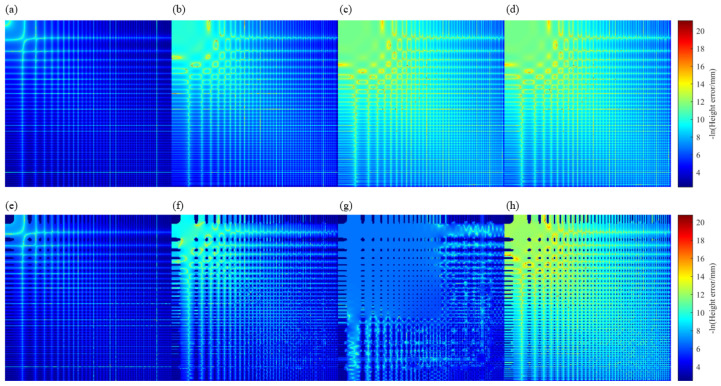
Reconstructed residuals: (**a**–**d**) errors in the TFLI, HFLI, SLI, SLIH method, respectively, with the complete data; (**e**–**h**) errors in the TFLI, HFLI, SLI, SLIH method, with the incomplete data.

Reconstruction errors are visualized using the negative logarithm of the absolute height error. As shown in Figure 14a, the TFLI method exhibits the highest error (RMS = $2.6\times 10^{-2}$ µm, PV = 0.19 µm), followed by the HFLI (RMS = $5.8\times 10^{-3}$ µm, PV = 0.17 µm), as shown in Figure 14b. The SLI and SLIH methods achieve significantly lower errors (RMS = $9.6\times 10^{-4}$ µm, PV = 0.03 µm), as shown in Figure 14c,d.

We then mask regions where the absolute slope exceeds 0.9 to increase the boundary discontinuities, as shown in Figure 14e–h. In this case, the TFLI and HFLI errors increase significantly. The truncation errors in the numerical calculations, especially in the high-frequency region, are the main source of the total errors (RMS = $2.5\times 10^{-2}$ µm, PV = 0.1893 µm) of the TFLI, followed by the HFLI (RMS = $1.1\times 10^{-2}$ µm, PV = 0.1344 µm), as shown in Figure 14e–h. The reconstruction error (RMS = $2.3\times 10^{-3}$ µm, PV = 0.0958 µm) of the SLI, while the SLIH method retains low error (RMS = $1\times 10^{-3}$ µm, PV = 0.0327 µm). Therefore, the SLIH method demonstrates superior robustness to boundaries discontinuities.

**Figure 15:**
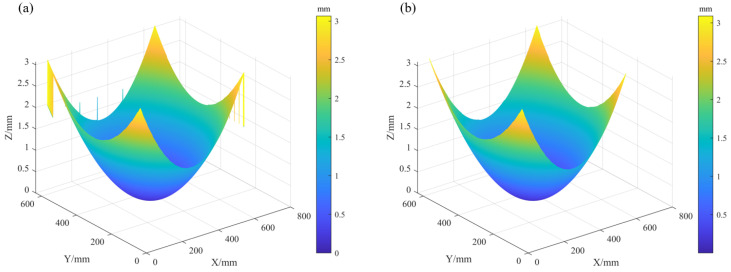

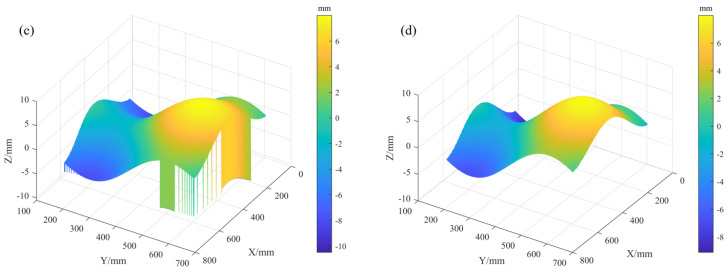
Edge artifacts in OC iterative reconstruction: (**a**,**c**) pronounced spikes appear at the edges of spherical and complex surfaces when HFLI is used; (**b**,**d**) the same artifacts are effectively eliminated with SLIH.

We then perform reconstruction using the HFLI and SLIH methods, and adopt the OC strategy. As can be seen from Figure 15a,c, when using the HFLI method, there is a height jump at the boundary of the SUT. However, due to the first-order continuity of Hermite interpolation at the boundary, the edge spikes on both the spherical and free-form mirror SUTs disappear in Figure 15b,d.

## 6. Experiment

### 6.1. System Setup and Characterization

**Figure 16:**
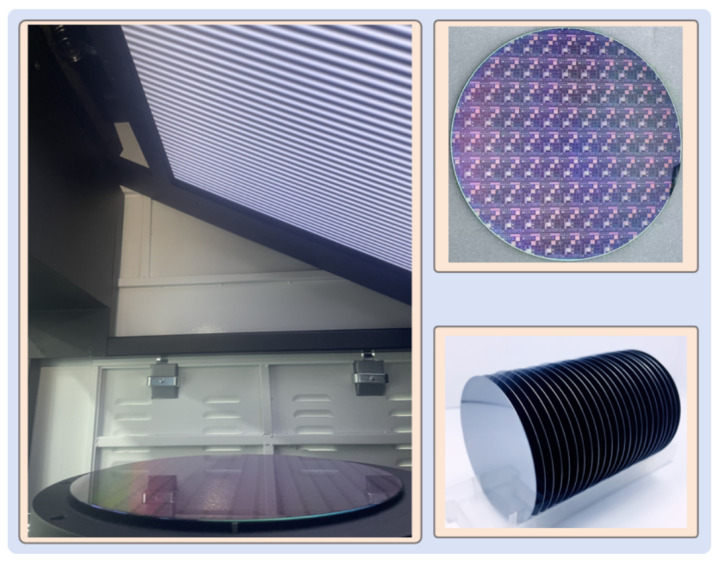
The Stress Mapping 500 (Model SM500) PMD system.

The PMD setup was assembled, as shown in Figure 16. The system incorporates an LCD panel (3840 × 2160 pixels, 0.2451 mm pixel pitch) and industrial cameras (MER2-230-168U3M, 1920 × 1200 pixels, 5.86 µm pixel size, Daheng Imaging, Beijing, China) equipped with 16 mm lenses. The wafer was horizontally positioned on a three-point support to define a stable reference plane.

**Figure 17:**
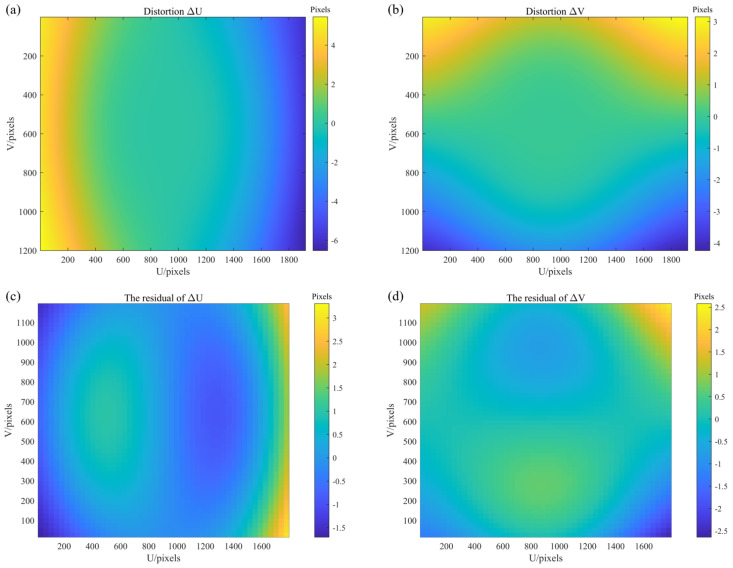
Distortion: (**a**,**b**) distortion maps in U and V directions under the radial model; (**c**,**d**) distortion maps in U and V obtained by reprojection bundle adjustment.

The main camera was calibrated using a checkerboard target, yielding a re-projection residual of 0.3 pixels, while the resulting distortion field shown in Figure 17a,b is primarily radial with values increasing toward the image margins.

Subsequently, four calibration datasets comprising twelve mirror poses in total were fed into the global optimization. When the conventional 2D reprojection residual was used as the cost function, the resulting camera distortion map deviated from the original calibration, exhibiting a saddle-shaped distribution in Figure 17c,d. This distribution, which shows greater distortion in the principal-ray region than in the margins, contradicts typical lens behavior and indicates that the optimization overfitted the camera model.

**Figure 18:**
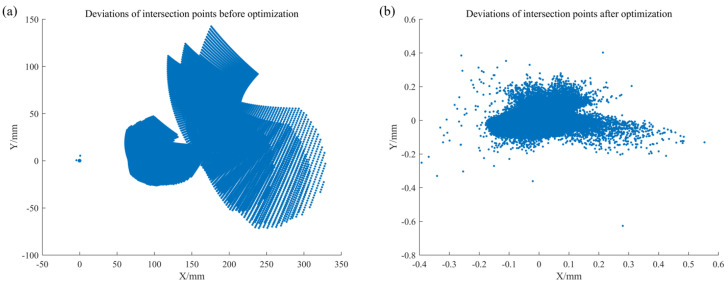
Backward ray-trace optimization: (**a**) initial residuals; (**b**) results after optimization.

We then carried out the optimization using the proposed backward-ray method. As shown in Figure 18a, the initial deviation between the ray-tracing intersection points and the phase-matched point exceeded 300 mm, revealing a large calibration error. However, after multi-pose optimization, the residual is reduced to 0.6 mm in Figure 18b, which is equivalent to roughly 3 screen pixels.

**Figure 19:**
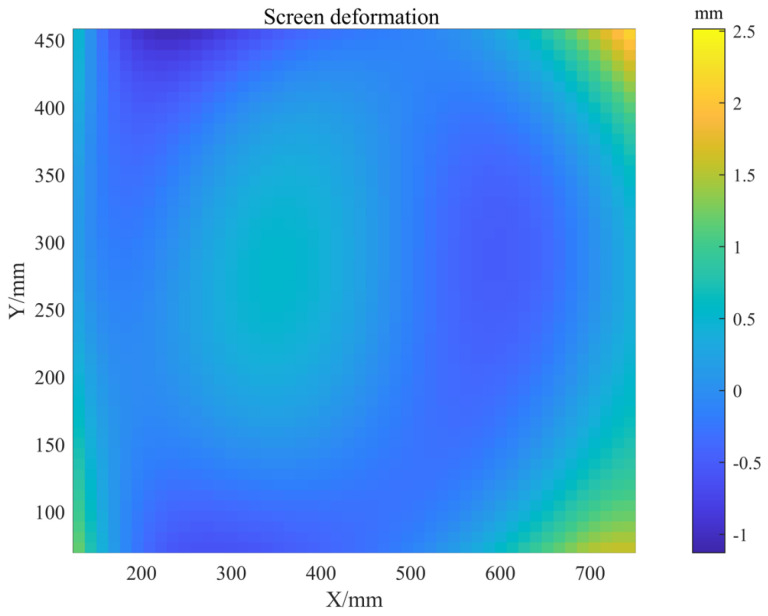
Fitted out-of-plane deformation of the screen.

The screen deformation was fitted with the proposed formula, and the result is shown in Figure 19. The PV deviation is 3.8 mm, which can no longer be neglected.

We then adopted the RC iterative strategy to conduct the reconstruction work, and compared the performance of two methods, including HFLI and SLIH. Meanwhile, we incorporated screen compensation parameters for comprehensive analysis. To determine the optimal solution suitable for wafer thin-film stress measurement, a spherical mirror with a nominal aperture of 150 mm and a concave radius of curvature of 500 mm was selected as the test object in the experiment. The measurement grid was configured with a 0.5 mm spacing.

**Figure 20:**
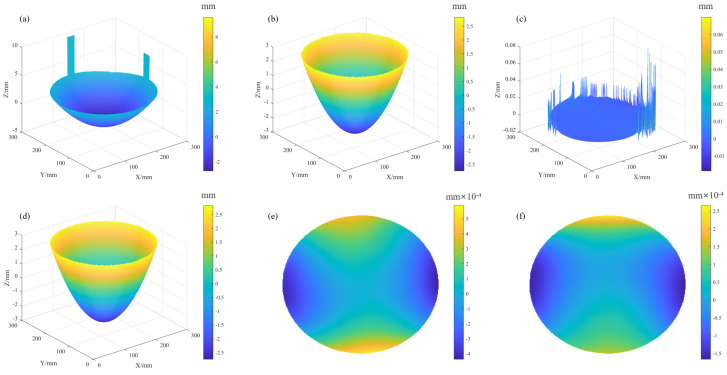
Reconstructed spherical surfaces: (**a**–**c**) HFLI reconstruction: the first iteration result, the filtered result after the fourth iteration, and the spherical fit residuals; (**d**–**f**) SLIH reconstruction: the fourth iteration result, the spherical fit residuals without screen compensation, and the spherical fit residuals with screen compensation.

The reconstruction results of this spherical mirror are shown in Figure 20. When the HFLI method was used for reconstruction, the first iteration produced pronounced edge spikes in Figure 20a, consistent with the simulation results. To obtain a usable reconstruction, the results after four iterations had to be filtered, as shown in Figure 20b. Although filtering reduced the amplitude of the spikes, the spherical fit residuals (radius = 495.7 mm, PV = 80.2 µm, RMS = 13.4 µm) still indicated severe edge artifacts, as shown in Figure 20c. When the SLIH method was used for reconstruction, no edge spikes appeared, as shown in Figure 20d.

We then evaluated the benefit of screen compensation in the reconstruction. The results after four iterations were fitted to a spherical surface. Without it, the resulting radius was 501.17 mm, with a PV error of 1.24 µm and an RMS error of 0.087 µm in Figure 20e. With compensation enabled, the radius converged to 500.59 mm, while the PV and RMS errors were reduced to 0.48 µm and 0.045 µm Figure 20f, respectively. This reduction in error demonstrates that the synergy between the proposed global optimization and the SLIH iterative reconstruction enhances both the stability and the ultimate accuracy of the measurement. Consequently, the final reconstructed sag of 5.65 mm for the curved mirror in Figure 20d further demonstrates the system’s capability to accurately measure warp within a 5 mm range at a precision of 0.5 µm.

### 6.2. Validation on Patterned and Bare Wafers

We then tested full-field curvature mapping on a 12-inch patterned wafer. Etching lowered the contrast in some regions, leaving missing data in the images. These voids were deliberately retained without any fitting or inpainting to avoid artificial results. Typically, the mean curvature is computed from the first fundamental form *E*, *F*, *G* and the second fundamental form *L*, *M*, *N* of the surface, as shown in Equation (14) [50].

(14) $$H=\frac{\left(E\times N+G\times L-2F\times M\right)}{2(E\times G-F^{2})}$$

**Figure 21:**
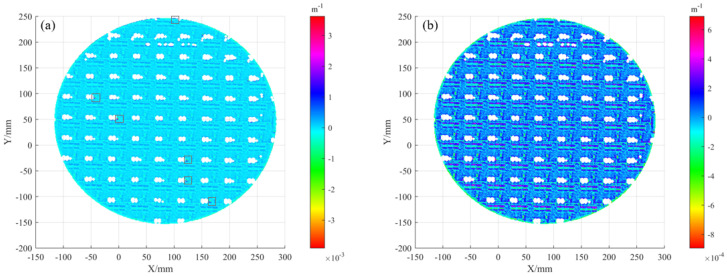
(**a**,**b**) Curvature maps of a 12-inch patterned wafer with incomplete data, obtained using the HFLI and SLIH methods, respectively.

As shown in Figure 21a, the HFLI method is compromised by complex boundaries due to missing data. This results in noticeable noise (highlighted in the boxes) and a failure to resolve local stress undulations from chip packaging. SLIH, by contrast, robustly handles these boundaries and clearly reveals the stress signature, making it the preferred choice for PMD-based chip-packaging inspection, as shown in Figure 21b.

**Figure 22:**
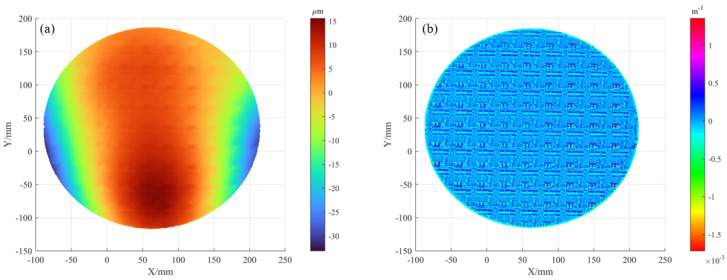
(**a**,**b**) Full-field warp and curvature maps of a 12-inch patterned wafer, respectively.

We then adjusted the exposure to achieve full-field warp inspection in Figure 22a. As shown in Figure 22b, the curvature distribution map reveals significantly elevated local curvature in the high-density logic cell regions, particularly at both edge areas. This increase is primarily attributed to residual stress induced by manufacturing processes such as the redistribution layer (RDL) [51]. Given that curvature is a direct measure of film stress, it serves as a critical criterion for in-line monitoring. Therefore, identifying dies with anomalous curvature enables effective quality control.

Finally, the proposed system was applied to full-field stress mapping of an 8-inch Si wafer (0.725 mm, $E_{s}$ = 169 GPa, $\nu_{s}$ = 0.25) coated with a 620 nm tungsten film.

**Figure 23:**
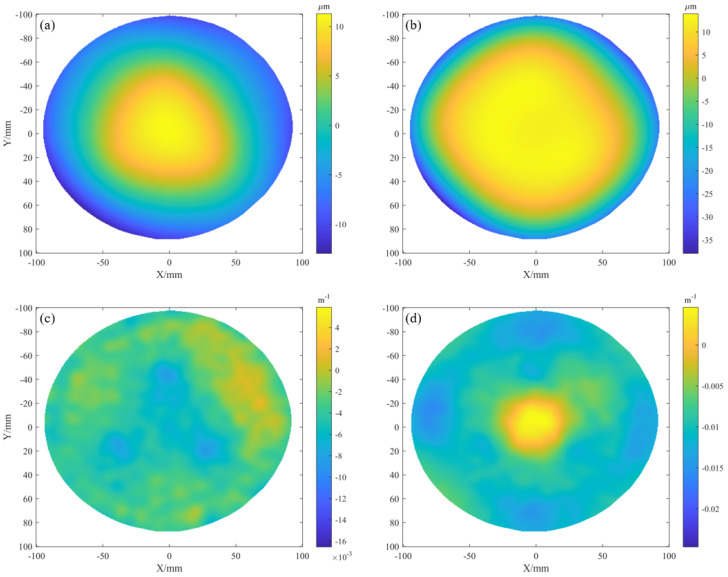
Results for the 8-inch wafer: (**a**,**b**) warp distributions before and after film deposition; (**c**,**d**) mean curvature distributions before and after film deposition.

Figure 23a,b show the wafer warp distribution before and after thin-film deposition. Specifically, Figure 23a displays the initial warp before deposition, representing the inherent topography of the substrate, while Figure 23b shows the warp after deposition, where the observed changes originate from stress introduced by the thin film. Figure 23c,d present the corresponding mean curvature distributions, quantifying the local bending degree of the wafer before and after deposition.

**Figure 24:**
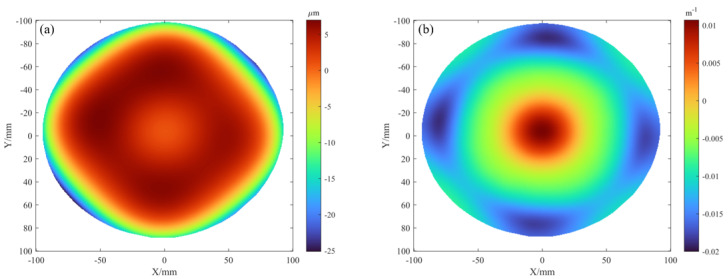
Deformation results for the 8-inch wafer: (**a**) warp deformation caused by the thin film, (**b**) mean curvature of the warp deformation.

Subsequently, Figure 24a shows the warp difference calculated from Figure 23a,b. This result effectively isolates the net effect of the thin film and thus directly represents the deformation induced solely by the film. Figure 24b presents the mean curvature difference derived from Figure 23c,d. This derived data serves as the essential input for subsequent thin-film stress extraction using the Stoney equation, completing the analytical pathway from deformation measurement to stress quantification.

To verify the accuracy of the measurement results in this study, we use the test results obtained from the mainstream industrial thin-film stress testing tool (Toho FLX-2320-S) as a reference. It should be noted that the PMD method enables full-field stress measurement, while the FLX device can only obtain stress data along two median lines (horizontal and vertical) of the wafer through scanning. Therefore, to ensure comparability with the FLX test results, when calculating the stress, we extracted the curvature data in both the x and y directions in Equation (15).

(15) $$K_{x}=\frac{L}{E},K_{y}=\frac{N}{G}$$

**Figure 25:**
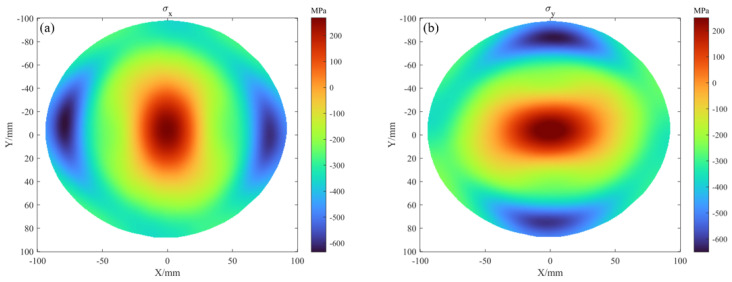
(**a**,**b**) represent the stress mapping, $\sigma_{x}$ and $\sigma_{y}$ of the 8-inch wafer, respectively.

The stress mapping $\sigma_{x}$ and $\sigma_{y}$ calculated using the Stoney formula is shown in Figure 25a,b.

**Figure 26:**
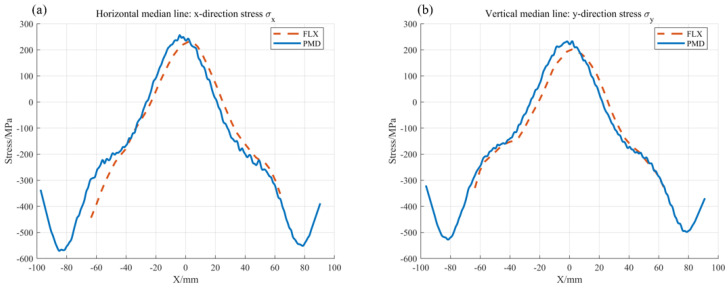
The measured thin-film stress is compared with the reference: (**a**) and (**b**) show the $\sigma_{x}$, $\sigma_{y}$ distributions on the horizontal midline and vertical midline of the 8-inch wafer, respectively.

For comparison, Figure 26a,b superimpose the FLX results (dashed lines) on the PMD-derived stress distributions (solid lines) from Figure 25. These solid line data correspond to the measurement paths of the FLX-2320-S, specifically referring to the stress distributions $\sigma_{x}$ in the horizontal direction and the $\sigma_{y}$ in the vertical direction.

Taking the central value as a reference, the $\sigma_{x}$ yields 230.5 MPa for FLX and 242.5 MPa for PMD in Figure 26a, while the $\sigma_{y}$ gives 206.6 MPa and 215.1 MPa in Figure 26b, resulting in deviations of 4.96 percent and 4.19 percent. The close agreement between the PMD-derived stress profile and the results from the sparse-data FLX fitting, mutually validating the reliability of the PMD method for wafer-level stress measurement. Moreover, PMD covers a larger area and clearly captures distinct inflection points at wafer edges in Figure 26a,b, whereas FLX cannot resolve these features. These results collectively demonstrate that the PMD method not only achieves high consistency with mainstream FLX equipment in terms of stress measurement accuracy but also can generate complete stress mapping, fully verifying its feasibility and significant advantages for wafer thin-film stress mapping.

### 6.3. Phase Error Analysis from Parasitic Reflections

When measuring thin films deposited on highly reflective substrates, reflections from both the front (film) and rear (substrate) interfaces may superimpose at the camera. This superposition may generate “ghosted” fringe patterns that introduce phase errors [52].

**Figure 27:**
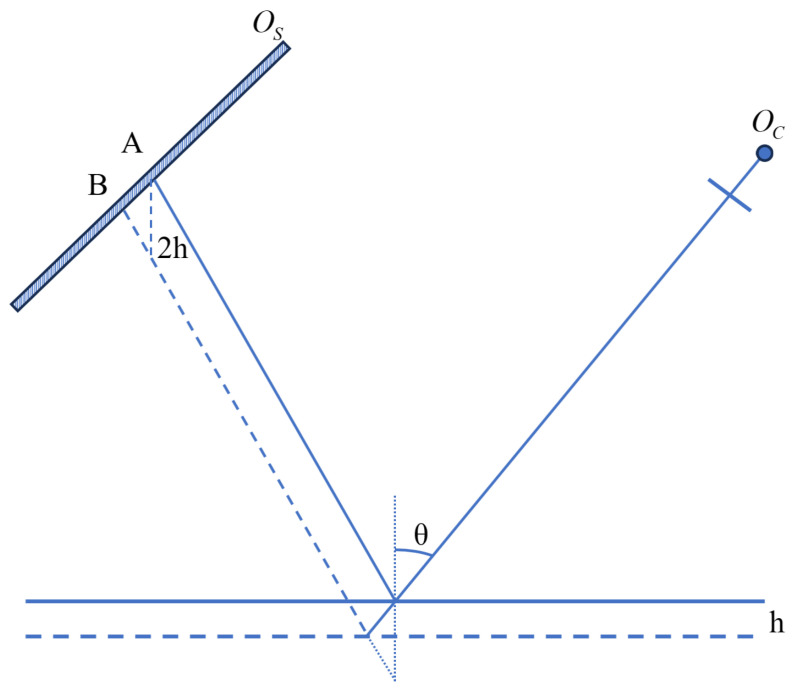
Superimposed fringe model.

The model of the parasitic reflections is shown in Figure 27. The intensity received at a camera pixel, resulting from the superposition of reflections from the two interfaces, can be expressed as Equation (16).

(16) $$I=I_{0}+M_{1}\cos\varphi_{A}+M_{2}\cos\left(\varphi_{A}+\Delta \varphi \right)$$

where $I_{0}$ is the background intensity, $M_{1}$ and $M_{2}$ are the modulation coefficients corresponding to the reflections from the front and rear surfaces, respectively. $\varphi_{A}$ is the phase of the primary (front-surface) reflection, and Δφ is the phase difference between the two reflected wavefronts. $\varphi_{A}$ and Δφ can be expressed as Equation (17).

(17) $$\left\{\begin{array}{c} \varphi_{A}=\frac{2\pi }{\Gamma \cdot p}\bar{O_{S}A}\\ \Delta \varphi =\varphi_{A}-\varphi_{B}=\frac{2\pi }{\Gamma \cdot p}\cdot \bar{AB} \end{array}\right.$$

where Γ is the fringe period and *p* is the pixel pitch of the screen, while $\bar{O_{S}A}$ and $\bar{AB}$ respectively denote the geometric distances from point *A* to the screen edge and between points *A* and *B*. As shown in Figure 27, the distance $\bar{AB}$ for a wafer of thickness *h* can be expressed by Equation (18), based on the triangle inequality.

(18) $$\bar{AB}<2h$$

Substituting Equation (18) into Equation (17) gives the maximum phase error in Equation (19).

(19) $$\Delta \varphi =\frac{4\pi h}{\Gamma \cdot p}$$

Equation (19) shows that the phase error is proportional to the substrate thickness and inversely proportional to the fringe period. Furthermore, Equation (16) can be expressed as Equation (20).

(20) $$I=I_{0}+M\cos\left(\varphi_{A}+\delta \right),$$

where *M* and δ are expressed as Equation (21).

(21) $$M=\sqrt{M_{1}^{2}+M_{2}^{2}+2M_{1}M_{2}\cos\Delta \varphi },\delta =\arctan\left(\frac{M_{2}\sin\Delta \varphi }{M_{1}+M_{2}\cos\Delta \varphi }\right)$$

When *h* < 1 µm, $M_{1}=M_{2}$, and a 32-pixel fringe period, the resulting error is insignificant ($\delta =8.01\times 10^{-4}$ rad). Therefore, given the nanoscale thickness of the film in wafer stress metrology, parasitic reflections can be effectively suppressed and rendered negligible when the fringe period exceeds 32 in the proposed system.

## 7. Conclusions

In this paper, a phase-measuring deflectometry (PMD) system is developed for full-field deformation and thin-film stress measurement on semiconductor wafers. The proposed system architecture avoids expensive interferometric components, potentially reducing capital investment compared to conventional solutions. The system employs an inverse bundle-adjustment method and adopts the reprojection constraint (RC) iterative strategy, utilizing Hermite zonal wavefront integration to achieve the desired measurement accuracy. Measurement results from a standard spherical mirror demonstrate that the system delivers a precision of 0.5 µm over a warp range of 5 mm. The system enables full-field curvature mapping of 12-inch patterned wafers, overcoming the limitations of sparse-data scanning techniques. This capability permits the quality assessment of individual dies instead of the entire wafer, facilitating finer-grained reliability evaluation. Stress measurements on an 8-inch bare wafer show less than 5% deviation from the industry-standard Toho FLX-2320-S instrument.

The combination of PMD with phase measuring profilometry (PMP) will be further explored to reconstruct wafer surfaces with mixed specular-diffuse reflective properties. Additional research will focus on developing high-speed PMD systems for real-time monitoring of dynamic processes such as thermal deformation under operational conditions. Moreover, robust methods will be developed to generate and resolve superimposed fringe patterns from both interfaces of thin films on coated wafers, enabling total thickness variation (TTV) mapping.

## Figures and Tables

**Table 1:** Comparison of PV (µm) results for different number of iterations.

Round	Spherical-RC	Spherical-OC	Free-Form-RC	Free-Form-OC
1	373.1	344.8	2206.7	1579.3
2	8.04	9.1	310.8	366.5
3	0.1	0.49	6.72	7.24
4	0.0916	0.35	0.2627	2.09
5	0.0915	0.348	0.2626	2.09

## Data Availability

The data presented in this study are included in the article. Further inquiries can be made available upon reasonable request.

## Appendix A

This appendix provides the mathematical definitions of the camera intrinsic matrix, camera extrinsic matrix, and mirror deformation parameters, which are utilized in the bundle adjustment optimization described in the Introduction.

### Appendix A.1. Camera Intrinsic Matrix

(A1) $$K=\left[\begin{array}{ccc} f_{x} & s & c_{x}\\ 0 & f_{y} & c_{y}\\ 0 & 0 & 1 \end{array}\right]$$

where $f_{x}$ and $f_{y}$ are the focal lengths (in pixels) along the *x* and *y* axes, respectively; $c_{x}$ and $c_{y}$ are the coordinates of the principal point (in pixels); and *s* is the skew coefficient (typically zero for modern digital cameras).

### Appendix A.2. Camera Extrinsic Matrix

(A2) $$\mathrm{RT}=\left[\begin{array}{cccc} r_{11} & r_{12} & r_{13} & t_{x}\\ r_{21} & r_{22} & r_{23} & t_{y}\\ r_{31} & r_{32} & r_{33} & t_{z}\\ 0 & 0 & 0 & 1 \end{array}\right]$$

where $r_{ij}$ are the elements of the rotation matrix R. Each row of R represents the unit-direction vector of a world coordinate axis expressed in the camera coordinate system; $t_{x},t_{y},t_{z}$ are the components of the translation vector t, denoting the coordinates of the world origin in the camera coordinate system.

### Appendix A.3. Mirror Deformation Parameters

(A3) $$p={\left[d_{x},\;d_{y},\;d_{z},\;n_{x},\;n_{y},\;n_{z}\right]}^{T}$$

where $d_{x},d_{y},d_{z}$ represent the distances from the coordinate origin (the camera center) to the mirror plane, and $n_{x},n_{y},n_{z}$ denote the components of the unit normal vector of the mirror, defining its orientation.
